# Measurement Properties of the Smartphone-Based B-B Score in Current Shoulder Pathologies

**DOI:** 10.3390/s151026801

**Published:** 2015-10-22

**Authors:** Claude Pichonnaz, Cyntia Duc, Nigel Gleeson, Céline Ancey, Hervé Jaccard, Estelle Lécureux, Alain Farron, Brigitte M. Jolles, Kamiar Aminian

**Affiliations:** 1 Haute Ecole de Santé Vaud (HESAV)//HES-SO, University of Applied Sciences Western Switzerland, Physiotherapy Department, Avenue de Beaumont 21, Lausanne 1011, Switzerland; E-Mail: Celine.Ancey@hesav.ch; 2 CHUV-UNIL, Orthopedics and Traumatology Department, Avenue Pierre-Decker 4, Lausanne 1011, Switzerland; E-Mails: Hervé.Jaccard@chuv.ch (H.J.); Alain.Farron@chuv.ch (A.F.); Brigitte.Jolles-Haeberli@chuv.ch (B.M.J.); 3 Laboratory of Movement Analysis and Measurement, Ecole Polytechnique Fédérale de Lausanne (EPFL), ELH 135 (Bâtiment ELH), Station 11, Lausanne 1015, Switzerland; E-Mails: cyntia.duc@a3.epfl.ch (C.D.); kamiar.aminian@epfl.ch (K.A.); 4 School of Health Sciences, Queen Margaret University, Edinburgh EH21 6UU, UK; E-Mail: ngleeson@qmu.ac.uk; 5 CHUV-UNIL, direction médicale, Rue du Bugnon 46, Lausanne 1011, Switzerland; E-Mail: Estelle.Lecureux@chuv.ch

**Keywords:** shoulder, shoulder function, measurement properties, outcome assessment, validation studies, smartphone sensors, body-worn sensors, kinematics

## Abstract

This study is aimed at the determination of the measurement properties of the shoulder function B-B Score measured with a smartphone. This score measures the symmetry between sides of a power-related metric for two selected movements, with 100% representing perfect symmetry. Twenty healthy participants, 20 patients with rotator cuff conditions, 23 with fractures, 22 with capsulitis, and 23 with shoulder instabilities were measured twice across a six-month interval using the B-B Score and shoulder function questionnaires. The discriminative power, responsiveness, diagnostic power, concurrent validity, minimal detectable change (MDC), minimal clinically important improvement (MCII), and patient acceptable symptom state (PASS) were evaluated. Significant differences with the control group and significant baseline—six-month differences were found for the rotator cuff condition, fracture, and capsulitis patient groups. The B-B Score was responsive and demonstrated excellent diagnostic power, except for shoulder instability. The correlations with clinical scores were generally moderate to high, but lower for instability. The MDC was 18.1%, the MCII was 25.2%, and the PASS was 77.6. No floor effect was observed. The B-B Score demonstrated excellent measurement properties in populations with rotator cuff conditions, proximal humerus fractures, and capsulitis, and can thus be used as a routine test to evaluate those patients.

## 1. Introduction

### 1.1. Measurement Properties in Shoulder Function Evaluation

The prevalence of shoulder pain is estimated at 26.9% [1]. This places the shoulder as the second most frequently affected body site behind the lower back. Despite the high occurrence of shoulder conditions, there is an on-going controversy about the best methods to evaluate the impact of pathologies on shoulder function. Numerous clinical questionnaires exist but the methodological and reporting quality of the validation studies is generally low [2]. As a consequence, none has been recognized as a universal standard [3,4,5]. Computerized movement analysis might be an alternative due to its precision and reliability. However, the use of computerized systems is restricted to research for reasons of cost, training, practicality, and accessibility. The use of smartphones allows these limitations to be largely overcome, as they are fitted with built-in movement sensors, working in three dimensions but are affordable and have become items of everyday life. However, the use of smartphones for scientific purposes requires prior scientific validation.

Clinicians and clinical researchers need thoroughly validated measurement methods to correctly evaluate the patient’s performance and the efficiency of therapeutic interventions. It is essential that the measurement properties of evaluation tools are extensively established to allow a correct interpretation of the outcome. In addition to the validity and the reliability, the investigation of the responsiveness and the definition of the clinically-important values are fundamental to correctly interpret the progress over time. This work requires a methodical process as the measurement properties are context-dependent. Thus, the investigations have to be performed in a large variety of situations to provide specific values for the clinicians to be able to tackle the wide range of conditions encountered in their practice [6].

Computer-based kinematic evaluation showed promising results for objective function evaluation but has remained too cumbersome for routine clinical application. Based on nine functional tests inspired from the Simple Shoulder Test (SST) [7], Coley *et al.* developed different scores (P, RAV, and M scores) using arm acceleration and angular velocity [8]. The kinematics have been recorded with arm-attached inertial sensors, with the aim to produce a valid and clinically-applicable kinematic score that can be straightforwardly performed in clinical settings. Recently the functional tests were simplified to provide a shoulder function score, named the B-B Score by including only two basic arm movements (hand to the Back + hand upwards as to change a Bulb) [9].

Considering the simplicity of the B-B Score and the inertial sensing facility provided by smartphones, the measurement of this score using a smartphone might make computerized shoulder evaluation much more accessible for clinicians and researchers. We have investigated the validity and the reliability of the shoulder function B-B Score measured with a smartphone in a preliminary phase of the present study. It was demonstrated that a smartphone produced comparable group measurements to an inertial sensor-based body-worn system [10]. However, the ability of the score to evaluate the patient’s progression and to differentiate the results according to pathologies have not been investigated yet. The responsiveness, minimal detectable change (MDC), minimal clinically important improvement (MCII), and patient acceptable symptom state (PASS) need to be evaluated to allow a well-substantiated interpretation of the results during the patient follow-up [11,12,13]. The MDC is the lowest value that can be considered as above the bounds of measurement error for an instrument [12]. The MCII is the smallest change in measurement that signifies an important improvement for the patient, and the PASS is the symptom state that the patients consider acceptable [11].

### 1.2. Influence of Shoulder Pathologies on Physiological Movement

The measurement properties for the B-B Score need to be determined first for conservatively-treated shoulder conditions, as they are much more frequent than surgically-treated conditions. Overall, only one in every sixteen patients presenting with shoulder pain requires surgery [14]. Moreover, some results were already available for the postsurgical context as the B-B Score was developed in a population who had undergone rotator cuff and arthroplasty surgery [9]. It has been established that the B-B Score produces comparable results to the kinematic P Score, which is valid and responsive following shoulder surgery [8,15,16].

Patients with rotator cuff conditions, proximal humerus fractures, adhesive capsulitis, and shoulder instabilities are frequently encountered in shoulder consultations [17,18,19,20,21,22]. It is, thus, essential to investigate the measurement properties of the B-B Score for these conditions. The validity and measurement properties of kinematic analysis may differ according to the type of pathology which affects the movement in a specific way. Thus, the B-B Score has to be validated separately for each pathology.

Conditions associated with the shoulder’s rotator cuff musculature are the most common source of shoulder pain (65%). They are caused by rotator cuff tendinopathy, rotator cuff tears, subacromial impingement or subacromial bursitis [23]. Rotator cuff tendinitis affects 29% of patients presenting with shoulder pain in general practice [19]. Rotator cuff tear prevalence is also very high and is strongly related to age. Tears are present in 2.5% of the general population in their 30 s, 25% in their 60 s, and 50% in their 80 s [18]. A painful arc during arm elevation is typical of rotator cuff conditions [24]. However, clinical presentation of rotator cuff conditions varies considerably. Range of motion (ROM) limitations may or may not be observed, and tears may remain asymptomatic despite the anatomical lesions [25].

Adhesive capsulitis, also named frozen shoulder, represents the second most prevalent cause of shoulder pain (22%) [18]. It is an idiopathic disease of the joint capsule causing mainly pain and stiffness [23]. The adhesive capsulitis is usually considered a 12- to 18-month self-limiting process, but mild symptoms may persist longer [26].

Proximal humeral fractures are also common, as they account for 6% of all adult fractures [20]. The incidence of this type of fracture in Western countries is growing due to the increasing age of the population. The movement is altered during the rehabilitation phase by pain, stiffness, and loss of strength. The recovery at one year is generally good for the conservative and the surgical approach [27].

Finally, the shoulder instability is also a frequent cause of medical consultation in younger populations. It is characterized by the inability to maintain the humeral head in the glenoid fossa of the scapula, so that the humerus slides partially or completely out of its socket. The shoulder instability’s one-year incidence is 0.56‰ individuals per year in the general population, but reaches 2.8% in a physically active young population [21,22]. Instability is problematic because it frequently leads to recurrent shoulder dislocation, apprehension, and loss of quality of life [28,29]. The movement is altered in the less stable positions of the glenohumeral joint. Typically, the patient experiences apprehension at the end of ROM while undertaking combined movements but can perform activities without problem in stable glenohumeral joint positions.

### 1.3. Study Aim and Hypotheses

This study is aimed at the determination of the measurement properties of the smartphone B-B Score for the assessment of the progression of current shoulder pathologies (rotator cuff condition, capsulitis, proximal humerus fracture, and shoulder instability).

Based on two assessments acquired over a six-month period, it was hypothesized that:

- the score would remain stable in the control group while it would progress significantly (*p* < 0.05) over time in each pathological group,
- the responsiveness would be comparable to that of validated clinical questionnaires,
- the area under the receiver operating characteristic (ROC) curve indicative of diagnostic power, would be at least adequate (≥0.70),
- the correlations with clinical questionnaires would be at least moderate (*r* > 0.50) [6,30].

No hypothesis was made about the MDC, MCII, and PASS values as these investigations primarily aimed at the determination of these values for the needs of clinical evaluation.

## 2. Experimental Section

### 2.1. Participants

A prospective cohort study was conducted between August 2011 and May 2014 at the Department of Traumatology and Orthopaedic Surgery of the University Hospital of Lausanne. Ethical approval was granted by the Human Research Ethics Committee of the Canton of Vaud (CER-VD). Patients gave their signed informed consent for the participation in the study.

Patients were adults (>18 years old). They presented with one of the following shoulder conditions, as stated during their first medical consultation at the specialized shoulder consultation unit of the hospital: a rotator cuff condition, shoulder instability, adhesive capsulitis, proximal humerus fracture. With the exception of patients with fractures, patients who gave their consent underwent a baseline measurement session within two weeks following the medical consultation, and a second session six months later. For patients with humerus fractures, measurements were performed six weeks post-stabilisation and six months later, provided that the radiological control showed normal healing.

Only patients who required conservative treatment were selected in the rotator cuff condition, capsulitis or instability groups. Patients undergoing surgical and conservative fracture treatments were included as the progress and functional prognosis is similar in both populations [27].

A group of participants younger than 35 years old without a history of shoulder condition/pain, was also included to evaluate the performance in a healthy population and the stability of the score. These participants were purposefully younger than the patients to avoid bias related to the high prevalence of asymptomatic rotator cuff tear above 40 years old [25].

The sample size calculation was based on the data of a pilot study that included seven controls and 16 patients. The calculation was made so that, with a significance level at *p* < 0.05, the power of 0.80 was reached when the minimal standards for acceptable properties of the score were met. Eighteen patients per group were needed for a significant correlation when *r* > 0.50, 11 patients for an area under a ROC curve of 0.80 with a standard error of 0.1, and nine patients for a significant difference between the patients and the control group [31,32]. According to these estimations, 20 participants were enrolled in each group of pathology and in the control group.

Exclusion criteria were a bilateral shoulder condition, any concomitant pain or condition involving the upper limb or cervical spine, medical contraindication to execute movements required for score completion, tumour, neurological conditions interfering with the test, and an insufficient local language level to give truly informed consent or to understand questionnaires.

### 2.2. Measurement Protocol Heading 

**Figure 1:**
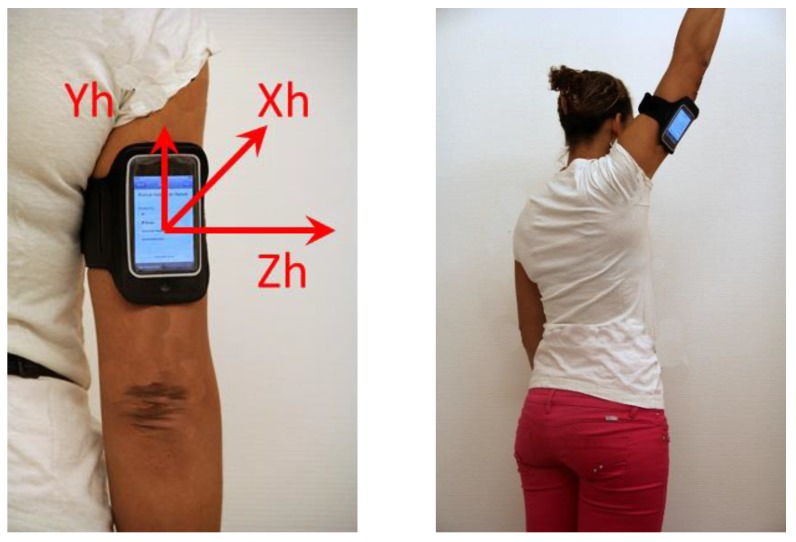
iPod ^®^ attached to the arm during the test completion.

Patients were measured using a smartphone (iPod^®^, Apple, Cupertino, CA, USA) attached to the back of the arm with an armband (Figure 1). The lower edge of the smartphone was set 3 cm above the upper edge of olecranon. The iPod was fitted with 3D built-in sensors (accelerometers: ±2 g precision: ±0.02 g; gyroscopes: ±500°/s precision: ±0.2°/s; sampling frequency: 100 Hz) [33].

After the setting-up of the system, the participants watched a video-recorded demonstration of the execution of the B-B Score. They were instructed to do the movements in the pain free ROM at their self-selected speed. Movements were executed in a standing position following smartphone-recorded instructions. The patients first undertook three repetitions of the two B-B Score movements on the healthy side (put hand to the back + hand to the ceiling as to change a bulb) and then repeated the task on the pathological side. The controls executed the same procedure beginning on the dominant side.

The B-B Score was computed as the ratio of a power-related unit [(deg/s) × (m/s^2^)] of the affected side relative to the healthy side, expressed as a percentage [8]. It was calculated along the method described in Pichonnaz [9].

**Figure 2:**
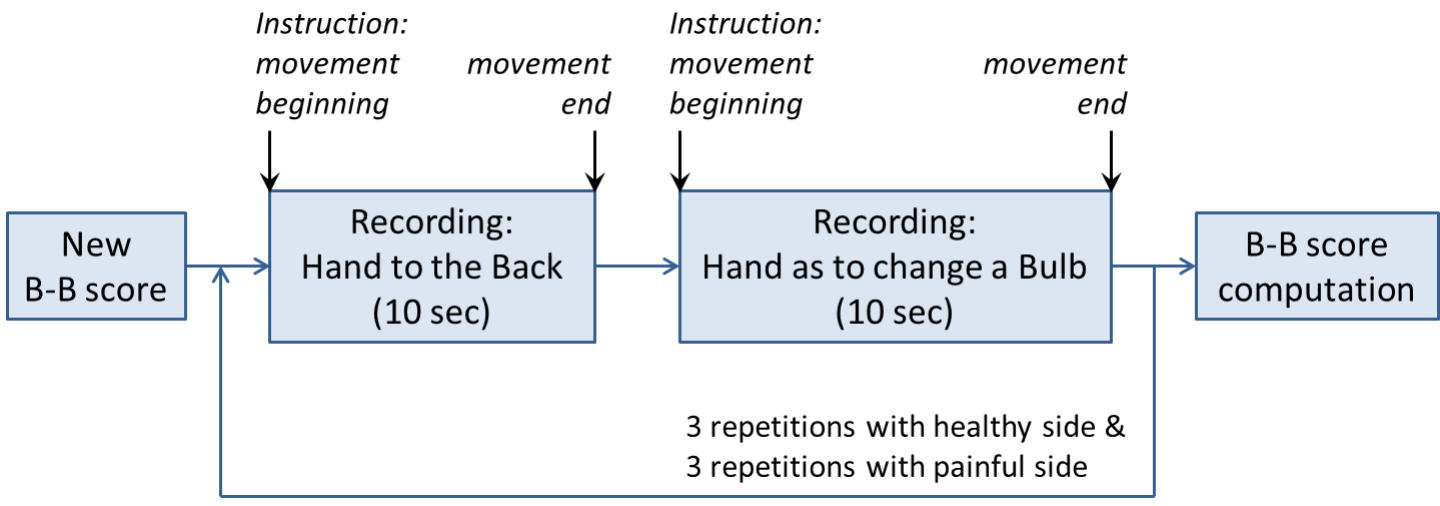
Schema of the application steps for the recording of a B-B Score.

An application, called iShould (instrumented shoulder test) was programmed in Objective-C [34,35]. This application enabled the acquisition of the acceleration and angular velocity signals during the movements of the shoulder, and the computation of the B-B Score value, as described in the Figure 2. Once the application had been initiated at the start of the assessment, the smartphone provided instructions to the user, through the smartphone loudspeaker, as to when the user should perform a movement associated with the B-B Score. For each score’ movement, the application recorded the acceleration and angular velocity signals for a predefined period of 10 s. The movements were first performed with the healthy side and then repeated with the painful side. At the end of the test, the B-B Score was directly calculated, displayed on the smartphone screen, and then stored on the smartphone. The application enabled exporting of all saved data to a computer for its direct comparison with the data from the inertial sensors of the reference system. 

One hundred percent represented a perfect balance between sides and the score decreases according to the severity of the functional loss. The score was calculated based on the mean over the three replications.

Clinical questionnaires were also completed. Four currently-used shoulder function questionnaires (Quick Disabilities of the Arm and Shoulder score (QuickDASH), Simple shoulder test (SST), Constant score and Constant relative score (based on an age-and sex-matched normal populations)), a specific shoulder instability questionnaire (Western Ontario Shoulder Instability Index (WOSI)), the EuroQol quality of life questionnaire (EQ-5D), and the pain visual analog scale (VAS) were completed [7,36,37,38,39,40]. The Constant Score was completed according to the modified guidelines [41]. The shoulder function questionnaires were selected because they represent current standards [5,42,43,44]. They allowed the evaluation of the concurrent validity for the B-B Score but not of its validity against a “gold standard”, due to the controversy surrounding shoulder function evaluation.

### 2.3. Analysis

Descriptive statistics were calculated for the patients’ characteristics and the outcomes at baseline and at six months. The differences between groups were analyzed using the Mann-Whitney or the chi-square tests as applicable, and the differences between stages were tested for each pathological group using the Wilcoxon signed rank test. The responsiveness for the baseline—six months evolution was calculated using Cohen’s *d* effect size with a 95% confidence interval. The diagnostic power for shoulder pathology detection was calculated using the ROC curve analysis. The area under the curve (AUC), sensitivity, specificity, and optimal detection threshold (highest sensitivity-specificity ratio) were calculated. The Spearman correlations were used to assess the strength of relationship between the B-B Score and the questionnaires for each of the pathologies. It was considered that a floor effect existed if >15% of patients scored less than 0 + MDC at baseline [13,45]. No ceiling effect was calculated as the score has theoretically no upper limit.

The MCII and PASS were determined for the patient group using the anchor-based method as described in Tubach *et al.* [11]. The MDC was calculated as described in Beaton *et al.* [12].

## 3. Results

One hundred and eight participants were tested at baseline (20 healthy participants, 20 patients with rotator cuff condition, 23 with fractures, 22 with capsulitis, and 23 with shoulder instability). All controls were measured at six months. Four patients could not be contacted at six months and four refused to participate for reasons without relationship with the study.

Drop-out rate was low (7%) and the number of patients lost at follow up were compensated to reach the planned sample size.

The population characteristics and the significance of the differences between groups are described in Table 1.

**Table 1:** Participants’ characteristics by group.

	Rotator Cuff (*n* = 20)	Fracture (*n* = 23)	Capsulitis (*n* = 22)	Instability (*n* = 23)	Control (*n* = 20)
Age mean (SD), Years	63.5 * (10.6)	60.1 * (15.6)	52.5 * (13.8)	32.1 (14.1)	28.2 (6.2)
Sex, % Women	50	78	60	43	50
Weight Mean (SD), kg	78.3 (18.2)	69.6 (15.1)	78.3 (15.1)	70.8 (12.9)	74.7 (17.4)
Body Mass Index Mean (SD), kg/m^2^	25.8 (5.4)	25.8 (5.4)	25.8 (5.4)	25.8 (5.4)	24.2 (3.9)
Size Mean (SD), m.	164.0 * (7.4)	167.7 (9.7)	172.4 (10.9)	172.6 (9.4)	175.0 (10.3)
Hand Dominance, % Right-Handed	90	87	100	87	90
Affected Side, % Dominant Side	70	25	45	52	-

* Significant difference with control group.

**Figure 3:**
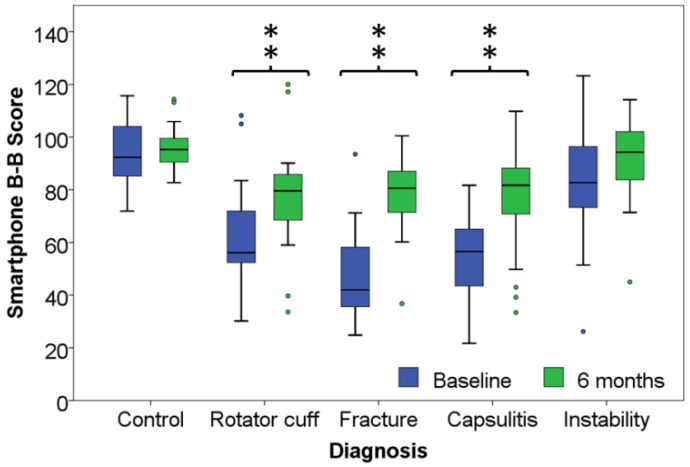
Outcome of the B-B Score for the control group and the pathology groups. **: significant difference with the control group *p* < 0.01.

The outcomes of the B-B Score for the control group, and for the patient group by pathologies are presented in Table 2 and Figure 3. The differences between the control group and the rotator cuff condition, fracture, and capsulitis patient groups were significant (*p* < 0.01). The difference between the shoulder instability group and the control group, was non-significant (*p* = 0.06).

**Table 2:** Mean and standard deviation of the B-B Score. Unit of scores are % representing the performance of the pathological side compared to the healthy side.

Pathology		Control	Rotator Cuff	Humerus Fracture	Capsulitis	Shoulder Instability
Baseline	Mean (SD)	94.1 (11.1) *	63.1 (19.7) *	46.3 (17.5) *	54.4 (14.6) *	84.5 (22.6)
	Sample size	20	20	23	22	23
6 months	Mean (SD)	96.0 (8.3) *	77.6 (21.1) *^,†^	78.9 (15.1) *^,†^	75.3 (20.5) *^,†^	91.2 (15.6)
	Sample size	20	19	20	21	20

* Significant difference with the control group (*p* < 0.01); ^†^ Significant difference with baseline (*p* < 0.01).

The effect size and 95% confidence intervals are presented in Table 3 for the B-B Score, Constant and Constant relative score, the SST, QuickDASH, and WOSI. The area under the curve (AUC) with 95% CI and the cut-off for optimal sensitivity-specificity ratio are detailed in Table 4. The correlations between the shoulder function questionnaires are presented for each pathologies in Table 5.

**Table 3:** Effect Size (95% CI) for each score and each pathology.

Outcome Measure	Rotator Cuff	Fracture	Capsulitis	Instability
	Effect Size (95% CI)			
B-B Score	0.69 (0.02–1.33)	1.94 (1.14–2.67)	1.16 (0.49–1.79)	0.10 (−0.52–0.72)
Constant	0.54 (−0.12–1.18)	2.09 (1.26–2.83)	1.05 (0.38–1.67)	0.21 (−0.42–0.82)
Relative Constant	0.50 (−0.15–1.14)	2.10 (1.27–2.84)	1.04 (0.38–1.67)	0.27 (−0.36–0.89)
SST	0.52 (−0.13–1.16)	1.65 (0.89–2.35)	0.86 (0.22–1.48)	0.10 (−0.53–0.71)
QuickDASH	0.35 (−0.30–0.98)	1.25 (0.53–1.91)	0.55 (−0.08–1.16)	0.01 (−0.61–0.63)
WOSI	-	-	-	0.47 (0.17–1.09)
EQ-5D	0.23 (−0.42–0.86)	0.76 (0.09–1.40)	0.34 (−0.27–0.94)	0.37 (−0.26–0.99)
EQ-5D VAS	0.07 (−0.57–0.70)	0.37(−0.26–0.99)	0.06 (−0.55–0.66)	0.11 (−0.51–0.73)

**Table 4:** ROC curve analysis results for classifying pathologies.

	**AUC** (95% CI)	**B-B Score** Threshold (%)	**Sensitivity** (%)	**Specificity** (%)
Rotator Cuff	0.90 (0.78–1.00)	83.6	90	90
Humerus Fracture	0.98 (0.94–1.00)	71.6	100	96
Capsulitis	0.99 (0.98–1.00)	82.1	95	100
Shoulder Instability	0.67 (0.50–0.84)	81.6	95	48

**Table 5:** Spearman correlation coefficients between B-B Score and clinical questionnaires.

	Rotator Cuff	Humerus Fracture	Capsulitis	Shoulder Instability
Constant	0.82 **	0.70 **	0.68 **	0.46 *
Relative Constant	0.84 **	0.69 **	0.69 **	0.43 *
SST	0.63 **	0.66 **	0.76 **	0.52 *
QuickDASH	−0.55 *	−0.40	−0.64 **	−0.57 **
WOSI	-	-	-	0.58
VAS pain	−0.50 *	−0.07	−0.39	−0.19
EQ5D	0.33	0.18	0.63 **	0.46 *
EQ5D-VAS	0.16	−0.30	0.44 *	0.47 *

SST: simple shoulder test; QuickDASH: Quick Disabilities of the Arm, Shoulder and Hand score; WOSI: Western Ontario Shoulder Instability Index; SSV: Subjective Shoulder Value; VAS: Visual Analog Scale. * significant correlation (*p* < 0.05); ** significant correlation (*p* < 0.01).

The MDC was 18.1%. The MCII of the B-B Score was 25.2% and the PASS was 77.6. No floor effect was observed as all patients performed above the MDC.

## 4. Discussion

This study aimed at the determination of the measurement properties of the smartphone B-B Score in current shoulder pathologies (rotator cuff conditions, capsulitis, proximal humerus fractures, and shoulder instabilities).

### 4.1. Results Interpretation

Participants younger than 40 years old were purposefully enrolled in the control group to prevent the inclusion of people with undetected rotator cuff conditions [25]. Thus, the significant difference in patient size between the rotator cuff group and the control group reflects the age-related decrease in size [46]. It is not likely to have an impact on this study’s results as age is not known to have an influence on symmetry in arm movement, as measured by the B-B Score. The high proportion of women in the fracture group is representative of gender prevalence in the wider population [20]. The low proportion of patients affected on the dominant side in the same group is of minor importance, as the outcome is not influenced by the fracture side [47]. Further, the influence of dominance on the B-B Score is minimal, as observed in the control group and in a previous study [9].

The B-B Score differences between the control and the patient groups were highly significant with the exception of the shoulder instability group. The functional loss was, in order of importance, more marked for patient with a fracture, a capsulitis, and a rotator cuff condition than for instability. Hence, the B-B Score clearly discriminated the three first groups from the healthy group but displayed a lower discriminative power for shoulder instability.

Shoulder instability is characterised by apprehension in the arm positions that exposes the patient to a glenohumeral dislocation risk [29]. It might be that the B-B Score is not challenging enough for these patients, as it is executed in the pain-free ROM and involved a self-chosen speed. Thus, the movement of the involved shoulder is not affected by the instability in the normal testing conditions of the B-B Score. Consequently, the functional loss may remain undetected. A more challenging version of the score inducing apprehension is hardly conceivable, as it might put the patient in a situation of actual dislocation likelihood. These results highlight that shoulder instability affects movement in a different way than other shoulder pathologies and should, thus, be evaluated using a specific tool, like the WOSI, for example.

The non-significant baseline to six-month progression in the control group indicated that the B-B Score is stable over time during which the participant’s performance can reasonably be expected to have remained unchanged. The significant differences over time observed in the rotator cuff condition, humerus fracture, and capsulitis groups indicate that the B-B Score discriminates amongst clinical stages for these pathologies. Conversely, no significant difference was found in the shoulder instability group.

It should be noted that the treatments were not standardized in this study as the aim was to evaluate the score properties but not the treatment’s efficacy. Thus, the observed results reflect the combination of the natural evolution and of the individualized treatment received by the patients.

The effect size measured in this study should be considered as indicative, as the confidence intervals were large. The effect sizes were larger, in order of importance, for the rotator cuff, humerus fracture, and capsulitis conditions, than for the shoulder instability condition. These results are essentially related to the respective baseline to six-month progression in each one of these pathologies. As a consequence, the absolute value of the effect size is relative to the context of measurement and, hence, the reference to cut-off values can be misleading [48].

Conversely, the comparison of the effect sizes of concurrent measurement methods for a given condition is informative towards the respective responsiveness of a score. The B-B Score was the most responsive score for the rotator cuff and capsulitis groups. The Constant and Constant relative score displayed the better responsiveness for humerus fracture, followed by the B-B Score. The B-B Score nevertheless constitutes a reasonable alternative to the Constant score for fracture evaluation, when the patient is unable to perform the strength measurement (as is the case in 51.9% of patients referred for shoulder surgery), and when the administrative burden is of concern [4]. All shoulder function evaluation methods showed better responsiveness than the EQ-5D generic quality of life questionnaire. No floor effect was observed for the B-B Score as all patients performed above the MDC value.

Similarly, to the Constant, DASH, and SST, the B-B Score demonstrated a poor responsiveness for shoulder instability. The WOSI displayed the best responsiveness for the evaluation of the shoulder instability. The limited responsiveness of the Constant, DASH, and SST for this patient population has previously been reported in the literature [40,49,50].

The AUC were excellent (≥0.90) for all pathologies except shoulder instability. The diagnostic power of the B-B Score was higher for fractures and capsulitis (0.98 to 0.99) than for rotator cuff conditions (0.90). The sensitivity and specificity at the optimal threshold were excellent for these three pathologies. Conversely, the diagnostic power was insufficient in the instability group as the AUC was lower than the 0.70 threshold [51]. Thus, the B-B Score is highly efficient for detecting loss of shoulder function in rotator cuff, fracture, and capsulitis. However, although the score is able to detect that pathology impairs shoulder function, it is not able to differentiate amongst pathologies. Further research should investigate to what extent alterations in specific movement patterns might allow discrimination amongst pathologies.

The correlations for the B-B Score with the Constant, Constant relative, and SST were moderate to high (0.63 to 0.82) for rotator cuff conditions, factures, and capsulitis [30]. In contrast, the relationship with the QuickDASH was generally lower (0.36–0.64) and non-significant in some cases. The merely objective nature of the B-B Score and the merely subjective nature of the QuickDASH may explain the lower relation with this questionnaire. The lower correlations with the VAS pain scale indicated that the B-B Score is essentially a measure of shoulder function.

Moderate to low correlations were found between the B-B Score and shoulder function questionnaires when considering instability. These results indicated that the relation to function was limited for this pathology. Conversely, the B-B Score adequately captured shoulder function for rotator cuff, fracture, and capsulitis. The absence of a floor effect indicated that the responsiveness was not altered for patients performing at a low functional level.

Some clinically useful values (MDC, MCII, and PASS) were also calculated in this study. No differentiation between pathologies was made due to the limited sample size. The MDC reflects the magnitude of change that is needed to consider that the change is greater than the measurement error for an instrument [12]. The MDC of the B-B Score using a smartphone indicated that the score difference needs to be greater than 18.1% to ensure that it is a real variation of a patient’s state. The MCII characterizes which level of score improvement reflects a meaningful progress for the patient [52]. Based on the MCII value, the B-B Score improvement between two stages needs to be greater than 25.2% for the patient to consider the improvement as meaningful. The PASS is the value beyond which patients consider themselves well [53]. Patients performing above the 77.6% will usually consider that the function loss is acceptable.

### 4.2. Limitations and Further Developments

Limitations are related to the limited sample size of each patient group. Though the group size was sufficient to compare the measurement properties of the B-B Score with those of concurrent scores, larger sample sizes would be needed to get more precise estimations. Additionally, the MDC, MCII, and PASS could not be calculated separately for each pathology group.

Though the B-B Score was compared to frequently-used shoulder function questionnaires, none of them is considered as a gold standard for shoulder function evaluation. Thus, the results of this study could solely investigate the concurrent validity but not the validity of the new score by comparison to a gold standard. The use of other questionnaires would have provided a different benchmark for the comparisons. It can nevertheless be considered that the questionnaires used in this study are fair comparators as no concurrent questionnaire has demonstrated its superiority over them [2].

The results found in this study demonstrated that the B-B Score has limitations for the evaluation of patients with shoulder instability. The score discriminated neither the instability from the control group, nor the stages within the instability group. Additionally, the responsiveness was lower than that of the WOSI and the diagnostic power was poor [54]. Based on these results, the B-B Score should not be used for the evaluation of shoulder function in a shoulder instability population. Conversely, all minimum requirements were met for rotator cuff conditions, proximal humerus fractures, and adhesive capsulitis.

Based on this study, it can be considered that clinically-important measurement properties of the smartphone-based B-B Score have been defined. The determination of the clinically useful values for the shoulder pathologies considered in this study provides a background for adequate interpretation of the results in research and clinics. However, a benchmark with a reference measurement system has not been provided in this study. Future studies should compare the results, reproducibility, and diagnostic power of a smartphone and a scientific measurement device. More research is also needed in patient populations that were not investigated in this study. For example, robust validation of the B-B Score is needed within populations experiencing glenohumeral osteoarthritis, shoulder arthroplasty, and rotator cuff surgery that have been the focus of validation studies in the past [9].

A middle segment smartphone model was chosen to have an insight into the performance of an accessible model. As a wide range of smartphones have similar or better quality sensors, the results from these models should, theoretically, be comparable to those found in this study. The B-B Score is probably robust to device variations, as it compares the performance of the affected shoulder with that of the healthy one. Thus, systematic errors in measurement affecting both sides will not affect the score. However, the influence of the characteristics of each smartphone on the outcome has to be investigated and quantified before clinical implementation.

The scientific value of a novel and objective test of shoulder function, the smartphone B-B Score technique, has been endorsed by the findings of this study, but no cost analysis was conducted at this stage of development. Further studies reproducing routine working conditions should evaluate this aspect. Given the reasonable material costs and the simplicity of the procedure, there would be a reasonable expectation for a favorable outcome following scrutiny by a formal cost-analysis.

Information and communication technologies developments were not considered in this study but may be possible at a later stage. The use of a smartphone makes the measurement much more accessible for clinicians or event patients. Thus, larger scale data collection could be performed by more evaluators at a lower cost. The smartphone B-B Score measurement might, for example, be used in telemedicine due to its simplicity and accessibility. It could also facilitate the centralization of data collected in a large number of settings at an acceptable cost, thus facilitating data collection for multicentric studies and registries.

## 5. Conclusions

The smartphone B-B Score demonstrated excellent measurement properties in populations with a rotator cuff condition, proximal humerus fracture, and capsulitis. The diagnostic and discriminative power were excellent for these populations. The correlations with the clinical questionnaires indicated that the B-B Score is valid for shoulder function evaluation. The responsiveness compared favourably with clinical questionnaires and no floor effect was detected. The determination of the MDC, MCII, and PASS provided a robust basis for the clinical interpretation of the outcome.

This opens interesting perspectives for routine objective shoulder function measurement in clinics, as this validated score can quickly be performed with an inexpensive device. The affordable measurement of large cohorts of participants may also be facilitated. However, the performance of the smartphones should first be compared to that of scientific measurement devices. Further investigation is also needed to devise a kinematics smartphone-based score for the evaluation of shoulder instability where the B-B Score did not meet the minimal requirements. Moreover, the measurement properties of the B-B Score should be further investigated in patient populations presenting other shoulder conditions. Studies could also explore the possibility to use the smartphone B-B Score for remote follow-ups and for early detection of suboptimal recovery.
